# An approach to quantifying the plausibility of the inadvertent download defence

**DOI:** 10.1080/20961790.2016.1253142

**Published:** 2016-12-16

**Authors:** Richard E. Overill, Kam-Pui Chow

**Affiliations:** a Department of Informatics, King's College London, London, UK; b Department of Computer Science, University of Hong Kong, Pokfulam, Hong Kong, China

**Keywords:** Forensic science, probability theory, confidence interval, binomial distribution, inadvertent download defence, possession of child pornography, counter terrorism, counter espionage

## Abstract

A table of 95% confidence limits on the probabilities for randomly downloading relatively small numbers of illegal images or sensitive documents amongst a relatively large number of other images or documents has been computed. It is anticipated that these data will assist prosecution officials in arriving at a decision as to whether or not there is a reasonable likelihood of a successful criminal prosecution when the inadvertent download defence is employed in cases of possession of child pornography, terrorist material or espionage-related documents. The same data can also be used by defence counsels to assess the strength of the prosecution's case.

## Introduction

Prosecutions for the possession of illegally downloaded digital material have caused serious problems for both digital forensic examiners and prosecution authorities alike for well over a decade. In previous studies based upon complexity theory [1–5] the plausibility of the Trojan Horse Defence [6–10] has been analysed. This defence has been successfully employed against prosecutions for the possession of large quantities child pornography (CP) images [11,12], but it also applies to images of terrorist material or espionage-related documents.

In this paper, we address another commonly offered defence against the possession of CP images or the illicit possession of other documents, when only a small proportion of the recovered downloaded material is illegal. One typical real-world example is the recovery of a relatively small number of CP images distributed amongst a much larger number of adult pornography (AP) or other non-CP images. In such a situation, the defence might claim that the defendant only intended to download the non-CP images, but because the website also happened to contain some CP images intermixed with the non-CP images, a few of the CP images were accidentally or unintentionally downloaded along with the intended non-CP images. This has been termed the inadvertent download defence (IDD) [13] and it has become a cause for serious concern amongst law enforcement and prosecution authorities in Hong Kong, the United Kingdom and elsewhere, due to the difficulty of establishing intentionality beyond reasonable doubt in such cases.

What credence can be given to a defence such as this? More specifically, what proportion of CP images needs to be present in the download in order for the prosecution to arrive at an assessment that there is a reasonable chance of securing a conviction by refuting the IDD, *ceteris paribus*? Put colloquially, “once is accidental, twice is coincidental, but more than twice is intentional.” In the present paper we attempt to address these questions by means of conventional probability theory applied to a downloading scenario which has been generalized from two recent criminal cases in the Hong Kong SAR of the P.R. China. A number of simplifying but not unrealistic assumptions regarding the browsing behaviour and the downloading context of the suspect have been employed as outlined below.

## Methods

Let the number of distinct downloaded CP images be *n_c_*, and the number of distinct downloaded AP or other non-CP images be *n_a_*. Thus, the total number of distinct downloaded images recovered is *n_d_* = *n_c_* + *n_a_*.

Since the precise contents of extraterritorial and potentially dark web hosted websites from which the downloads were made cannot in general be investigated by local law enforcement officers, it is necessary to use random sampling theory to relate the proportion of CP images in the download to the proportion of CP images in the website as a whole. That is, for randomly selected images, the download contents are a representative sample of the website contents within some confidence interval that reflects the sampling error. This scenario is analogous to the classical problem described by Jacob Bernoulli [14] of sampling a sequence of *n_d_* balls each of which may be either black or white from an opaque urn containing an exceedingly large number of such balls, without replacing them [15]. The present context with non-replaced balls of just two colours entails the application of the binomial distribution, provided that the total number of balls in the urn is exceedingly large. By the law of large numbers the proportions in the sample converge on the proportions in the population as *n_d_* →∞, at a rate proportional to *n_d_* ^−1^ using the Markov concentration inequality [16]. Provided that the population size is so exceedingly large as to be effectively infinite then as the sample size approaches the population size, the proportions in the sample approach the proportions in the population asymptotically; larger sample sizes yield smaller sampling errors.

[17], which is itself based on earlier work [18–20], indicates how a numerical estimate of the confidence limits of a binomial distribution may be obtained from linear interpolation in tables of cumulative binomial terms. However, for the present application we proceed analytically, then numerically, as follows.

For a specified confidence level α, we wish to calculate the probability bounds that precisely *n_c_* distinct CP images are present amongst the *n_d_* distinct downloaded (sampled) images. For this purpose we assume that the website to be organized in such a way that any CP images are distributed randomly amongst the non-CP images (rather than in a special section, for example), so that the website owner could plausibly deny all knowledge of the presence of any CP images on their website. We consequently assume that the defendant encountered the CP images randomly whilst browsing the website contents and selected the image thumbnails for downloading as an integral part of their browsing activities.

Within the context of the model outlined above, the (unknown) number *N* of distinct images available for downloading from the website is considered to be so exceedingly large as to be effectively infinite, so that the probability of selecting either a CP image or a non-CP image remains essentially unchanged as the downloading (sampling) process proceeds. We term this the “infinite scenario”. Websites containing a (known) finite number of downloadable images *N* can also be modelled parametrically in a corresponding “finite scenario” and as *N̂* → ∞ the “finite” scenario approaches the “infinite” scenario asymptotically [13].

Since the observed probabilities of selecting a CP image and a non-CP image both remain sensibly constant at *ˆp* = (*n_c_* /*n_d_*) and *ˆq* = (*n_a_* /*n_d_*) = (1 − *ˆp*) respectively, the binomial theorem can be applied directly, and the probability of selecting a CP image lies in the interval *p*_1_ < *p* < *p*_2_ with some confidence level α.

In order to determine the confidence interval for *ˆp*, it is necessary to integrate the corresponding binomial term such that:

$$\int_{0}^{p_{1}}\left(\begin{array}{c} n_{d}\\ n_{c} \end{array}\right)\;p^{n_{c}}q^{n_{a}}dp\;=\;½\left(1-\alpha \right)/(n_{d}+1)$$

and

$$\int_{p_{2}}^{1}\left(\begin{array}{c} n_{d}\\ n_{c} \end{array}\right)\;p^{n_{c}}q^{n_{a}}dp\;=\;½\left(1-\alpha \right)/(n_{d}+1)$$

where $\left(\begin{array}{c} n_{d}\\ n_{c} \end{array}\right)$ represents the number of different ways of selecting *n_c_* distinct objects from *n_d_* objects; it is to be understood that the confidence interval is centrally located in the range [0, 1] with equal areas of ½ (1 − α) in both the left and right tails, and also that each of the (*n_d_* + 1) terms of the binomial expansion contributes precisely (*n_d_* + 1)^−1^ to the overall normalization of the binomial distribution.

Integrating by parts repeatedly and solving the resulting recurrence relations yields:

$$\int_{0}^{p_{1}}\left(\begin{array}{c} n_{d}\\ n_{c} \end{array}\right)\;p^{n_{c}}q^{n_{a}}dp\;=\;\frac{{1-q}_{1}^{n_{d}+1}}{n_{d}+1}-\;\left(\begin{array}{c} n_{d}\\ n_{c} \end{array}\right)\left\{\frac{p_{1}^{n_{c}}q_{1}^{n_{d}-n_{c}+1}}{n_{d}-n_{c}+1}+\sum_{i\;=\;1}^{n_{c}-1}\frac{\prod_{j\;=\;0}^{i-1}n_{c}-j}{\prod_{j\;=\;0}^{i}n_{d}-n_{c}+j+1}p_{1}^{n_{c}-i}q_{1}^{n_{d}-n_{c}+i+1}\right\}$$

and

$$\int_{p_{2}}^{1}\left(\begin{array}{c} n_{d}\\ n_{c} \end{array}\right)\;p^{n_{c}}q^{n_{a}}dp\;=\frac{q_{2}^{n_{d}+1}}{n_{d}+1}\;+\;\left(\begin{array}{c} n_{d}\\ n_{c} \end{array}\right)\left\{\frac{p_{2}^{n_{c}}q_{2}^{n_{d}-n_{c}+1}}{n_{d}-n_{c}+1}+\sum_{i\;=\;1}^{n_{c}-1}\frac{\prod_{j\;=\;0}^{i-1}n_{c}-j}{\prod_{j\;=\;0}^{i}n_{d}-n_{c}+j+1}p_{2}^{n_{c}-i}q_{2}^{n_{d}-n_{c}+i+1}\right\}$$

In order to determine the values of *p*_1_ and *p*_2_ that satisfy the specified confidence level α, the Method of Bisection or *Regula Falsi* [21] can be used to optimize *p*_1_ in the monotonically increasing interval [0, *ˆp*] and similarly to optimize *p*_2_ in the monotonically decreasing interval [*ˆp*, 1]. The confidence interval for the sampled proportion *ˆp* is then expressed as the confidence interval [min(*P*_1_, *P*_2_), *P̂*] for the unimodal probability *P* that precisely *n_c_* distinct CP images are present amongst the *n_d_* downloaded (sampled) images.

From a computational perspective, intermediate numerical values of exceedingly large (>>10^+300^) and exceedingly small (˂˂10^−300^) magnitudes inevitably occur during the computation of the probabilities for real-world scenarios, and these were handled by employing adaptive numerical scaling techniques to avoid overflow and underflow exceptions. The numerical stability of the probabilities obtained was also verified by performing each probability calculation in three distinct ways: namely, by computing the required products in ascending order, in reverse order and in symmetric pairwise fashion.

## Results

In order to make this model straightforward to use by law enforcement officers and legal counsel involved in actual criminal prosecution and defence cases, a table of the 95% confidence limits on *P̂* corresponding to a range of typical values for both *n_c_* and *n_d_* has been computed and can be found in Table 1. The values are rounded to four decimal places (i.e. to 0.01%). Within an adversarial legal system the lower confidence limit represents the “best case” result for the prosecution side and the “worst case” result for the defence side; *mutatis mutandis*, the upper confidence limit represents the “worst case” result for the prosecution side and the “best case” result for the defence side. Thus, both legal teams can obtain a quantitative indication of the probative value or plausibility of their respective cases from these data. To be explicit regarding the relationship between plausibility and probability, probabilities signify the quantities that define a particular monotonic scale on which degrees of plausibility can conveniently be measured [22]. We note that the confidence intervals for the sampled proportion *ˆp* computed by the present approach are significantly tighter than those produced by the “exact” Clopper–Pearson approach utilizing *Β* (Beta) or *F* (Fisher) distributions which are conservative in the sense that they are always greater than or equal to the true confidence interval; for example, the width of the confidence interval for *ˆp* in the case *n_d_* = 10, *n_c_* = 1 is just 88% of the width of the corresponding Clopper–Pearson confidence interval [23].

**Table 1.** 95% confidence limits on the probability *P̂* for the inadvertent random downloading of *n_c_* illicit images amongst *n_d_* images within the IDD model.

*n_d_*/*n_c_*	1	10	100	1000	10000	100000
1	―	―	―	―	―	―
10	0.0343―0.3874	―	―	―	―	―
100	0.0224―0.3697	0.0145―0.1319	―	―	―	―
1000	0.0244―0.3681	0.0127―0.1257	0.0055―0.0420	―	―	―
10 000	0.0207―0.3679	0.0131―0.1252	0.0056―0.0401	0.0019―0.0133	―	―
100 000	0.0149―0.3679	0.0131―0.1251	0.0039―0.0399	0.0019―0.0127	0.0006―0.0042	―
1 000 000	0.0149―0.3679	0.0131―0.1251	0.0039―0.0399	0.0019―0.0126	0.0005―0.0040	0.0002―0.0013

We now present two illustrative examples in which the methodology described above can be applied to the evaluative assessment of the plausibility of the IDD; the data are derived from two actual criminal cases from the Hong Kong SAR of the P.R. China [13].

*Case 1*: In District Court Criminal Case No. 968/2010, the defendant had over 30000 image files which he had downloaded on various occasions. Amongst them, there were 63 still images and 185 video clips, all 248 of which image files were of CP. The remainder were indecent and obscene materials, plus cartoons and comic story books. Here, *n_c_* = 248 and *n_d_* = 30000; we calculate that the 95% confidence interval lies between 0.0254 and 0.0029. That is, we can say with 95% confidence that there is a chance of no more than 2-½% that the recovered download was the result of the oblivious random browsing activity typified by the IDD.

*Case 2*: In District Court Criminal Case No. 32/2013, the defendant had 714430 image files (including still images and video clips) which he had downloaded on various occasions. Amongst them, there were 84 video clips which were of CP. The remainder were indecent and obscene materials. Here, *n_c_* = 84 and *n_d_* = 714430; we calculate that the upper 95% confidence limit lies at 0.0435. That is, we can say with 95% confidence that there is a chance of less than 4-½% that the recovered download was the result of inadvertent random browsing activity.

In the light of our earlier discussion of the convergence behaviour of the sampling error, it is worth remarking that our results demonstrate the expected tightening of the 95% confidence interval bounds with increasing sample size. It should also be mentioned that a single trivial modification to the software enables the computation to be carried out at any other confidence level (e.g. 99%).

## Discussion

It is important to emphasize the vital role that digital forensic metadata plays in interpreting the confidence limits in Table 1. In both of the actual criminal cases cited above, the image files were in fact downloaded over a substantial period of time, involving a number of separate downloading sessions accessing more than one website. Careful analysis of the creation, last modification and last access time and date stamps of the downloaded files is capable of revealing whether all the illicit files were downloaded during one session or from a single website, or whether their downloading was distributed across many sessions and/or websites. By focussing only on those sessions during which illicit files were downloaded, a different perspective on the defendant's downloading behaviour may emerge which may in turn require a revision of the sampled proportion and its confidence limits.

A second important role for digital forensic metadata is to provide answers to vital questions such as the following: is there evidence that each or any of the illicitly downloaded files was in fact accessed, copied or viewed? If analysis of the creation, last modification and last access time and date stamps of the downloaded files indicates that none of these activities actually took place then the defendant may be in a position to credibly assert that they were unaware not only of the contents of these files, but also of their very existence. This would greatly weaken the prosecution's case in establishing the *mens rea* (“guilty mind”) of the defendant with regard to possession of the material.

It is now common for consumers of illicit materials to use “in private” browsing in order to minimize the amount of web-related metadata (cookies, browsing history, etc.) to be found on their client machines. In addition, use of Tor and similar browsers results in minimal useful metadata at their internet service provider (ISP) and beyond into the dark web. It is therefore essential to extract the maximum possible information from the metadata mentioned above in order to understand as fully as possible the *modus operandi* of the suspect with regard to how the downloaded material was treated. Nevertheless, in some cases less cautious suspects will leave ample traces of their browsing and downloading activities, even including online search terms; however, the majority of suspects, if they use online search terms at all, will employ covert (rather than overt) search terms in order to obfuscate their true intentions and render it more difficult to make use of as convincing evidence in a court of law.

A final issue which digital forensic analysis is only beginning to address relates specifically to cases of suspected possession of CP images: is the subject of each of the images clearly identifiable as a minor (i.e. less than 16 years of age)? For subjects close in age below this boundary, defendants may attempt to credibly assert (perhaps on the basis of a low resolution thumbnail image) that they reasonably believed the subject of the image in question to be legally an adult. Despite substantial technical advances, current digital facial aging software [24] and human skin tone/texture recognition software [25,26] are not yet sufficiently reliable for this purpose, which means that for the time being the distressing task of “eyeballing” suspected CP images must continue to be carried out by human inspection.

## Conclusion

A table has been computed of the 95% confidence limits on the probabilities *P* for the downloading small numbers of illicit files amongst a proportionately much greater number of non-illicit files, under the assumptions of random illicit file distribution, browsing and downloading. One purpose of the tabulated data is to assist both prosecution authority officials and also defence counsels in evaluating the plausibility of the IDD which may be raised in cases where the proportion of illicit files in the download is small. As such, it is intended to aid officials of the prosecution authority in coming to an informed decision as to whether there is a realistic prospect of a successful criminal prosecution in any particular case, *ceteris paribus*. At the same time, however, it is also a tool that the defence side can employ in arriving at a view on the likely effectiveness of running the IDD in any particular case. The potential applications to criminal cases involving not only possession of CP images but also counter terrorism and counter espionage scenarios have also been outlined. Last, but by no means least, the importance of using digital forensic metadata to provide solid, reliable supporting evidence in such cases has been emphasized.
